# Hemodynamics in pulmonary arterial hypertension (PAH): do they explain long-term clinical outcomes with PAH-specific therapy?

**DOI:** 10.1186/1471-2261-10-9

**Published:** 2010-02-22

**Authors:** Peter Steele, Geoff Strange, John Wlodarczyk, Brad Dalton, Simon Stewart, Eli Gabbay, Anne Keogh

**Affiliations:** 1 Department of Cardiology, Royal Adelaide Hospital, Adelaide, SA, Australia; 2 Department of Epidemiology and Preventative Medicine, Monash University, Melbourne, VIC, Australia; 3 John Wlodarczyk Consulting Service, New Lambton, NSW, Australia; 4 School of Human Life Sciences, University of Tasmania, Launceston, TAS, and medScript, Canberra, ACT, Australia; 5 Baker Heart Research Institute, Melbourne, VIC, Australia; 6 Western Australian Lung Transplant Unit and Pulmonary Hypertension Service, Royal Perth Hospital, Perth, WA, Australia; 7 Victor Chang Research Institute, St Vincent's Hospital, Sydney, NSW, Australia

## Abstract

**Background:**

Pulmonary arterial hypertension (PAH) has witnessed dramatic treatment advances over the past decade. However, with the exception of epoprostenol, data from short-term randomized controlled trials (RCTs) have not shown a benefit of these drugs on survival. There remains a need to differentiate between available therapies and current endpoint responses which in turn, could be used to guide treatment selection and provide long-term prognostic information for patients.

**Methods:**

We performed a systematic literature search of MEDLINE and EMBASE databases for RCTs of PAH-specific therapy published between January 1980 and May 2009. Articles were selected if they contained a placebo comparator and described hemodynamic changes from baseline. We applied the weighted mean change in hemodynamic variables to the equation developed by the National Institutes of Health (NIH) Registry to estimate long-term survival with each therapy.

**Results:**

Ten RCTs involving 1,635 patients met the inclusion criteria. Suitable hemodynamic data were identified for bosentan, sitaxentan, sildenafil, epoprostenol, beraprost and treprostinil. 77.6% of patients were female and the mean (SD) age was 46.5 ± 4.9 years. 55.5% of patients had idiopathic PAH (iPAH), 23.9% PAH related to connective tissue disease, and 18.2% PAH related to congenital heart disease. Based on the effects observed in short-term trials and, relative to placebo, all analyzed therapies improved survival. The estimated 1-year survival was 78.4%, 77.8%, 76.1%, 75.8%, 75.2%, and 74.1% for epoprostenol, bosentan, treprostinil, sitaxentan, sildenafil, and beraprost, respectively. These estimates are considerably lower than the 1-year observed survival reported in several open-label and registry studies with PAH-specific therapies: 88% - 97%.

**Conclusion:**

When applied to the NIH Registry equation, hemodynamic changes from baseline appear to underestimate the survival benefits observed with long-term PAH therapy.

## Background

Pulmonary arterial hypertension (PAH) is a progressive disease, characterized by sustained elevations in pulmonary arterial pressure (PAP), increased pulmonary vascular resistance (PVR) with progression to right-sided heart failure and ultimately death [1]. A number of drug classes have been approved for this indication on the basis of randomized controlled trials (RCTs), namely prostanoids (epoprostenol and iloprost), a phosphodiesterase-5-inhibitor (sildenafil), and endothelin (ET)-receptor antagonists (ETAs) (bosentan, sitaxentan, and ambrisentan). However, to date epoprostenol is the only agent to have demonstrated a short-term survival benefit compared to patients treated with conventional therapy [2]. Unfortunately, the use of epoprostenol is complicated by infections, pump malfunctions, and the inconvenience of a permanent delivery system [3].

Of the other PAH-specific therapies, there is no consensus about the most appropriate agent. All of the available drugs display similar improvements in functional class and six-minute walk distance (6MWD) in 12-16 week trials. Given the paucity of data on which to differentiate the available therapies, there are continuing discussions as to the prognostic accuracy of surrogate endpoints such as cardiopulmonary hemodynamics. In 1991, D'Alonzo et al. identified that mortality in patients with PAH was closely associated with right ventricular hemodynamic function [4]. The authors developed a predictive equation based on mean right atrial pressure (mRAP), mean pulmonary artery pressure (mPAP) and cardiac index (CI). The accuracy of the equation and predictive nature of the three hemodynamic variables were subsequently validated by several authors [5-7].

On the basis of this information, it seems both intuitive and logical to compare the degree of hemodynamic benefit provided by the available PAH-specific therapies, and to investigate whether these benefits can be used to explain long-term clinical outcomes in patients with iPAH. This information may be more clinically useful, provide a more accurate picture of patient prognosis, and present a clinical hierarchy in terms of surrogate endpoints that guides treatment selection based on the weight of available evidence.

In this report, we provide a systematic review of the literature with respect to changes in cardiopulmonary hemodynamics with PAH-specific therapy. Furthermore, we present new data, calculating the expected long-term benefit of therapies based on short-term hemodynamic changes.

## Methods

### Literature search

We searched MEDLINE and EMBASE databases for RCTs of PAH specific therapy published in English between January 1980 and May 2009. Candidate articles were selected by combining search results generated with exploded MESH subject headings or key words: 'hypertension, pulmonary' OR 'pulmonary hypertension' OR 'pulmonary arterial hypertension'; haemodynamics OR hemodynamics; bosentan OR sitaxentan OR sitaxsentan OR iloprost OR epoprostenol OR sildenafil OR ambrisentan OR beraprost OR trepostinil OR tadalafil OR vardenafil. The search strategy was limited to articles concerning human subjects, using placebo as the comparator, and accompanied by an abstract. Screening of titles and abstracts then identified potentially applicable studies. In addition, we searched the reference lists of included articles, practice guidelines and systematic reviews to identify relevant trials missed by the search strategy.

Two review authors independently selected articles for inclusion. Differences were resolved by discussion. Articles were selected that described hemodynamic changes from baseline and considered trials among patients with iPAH, or PAH associated with connective tissue diseases (CTD), congenital heart disease (CHD) or Human Immunodeficiency Virus (HIV). Only studies that assessed hemodynamics by right heart catheterization (RHC) were included. Articles were required to contain at least 10 patients in each arm, a minimum follow-up period of three months, and hemodynamic data pertaining to CI, mPAP and mRAP.

We excluded trials of PAH associated with chronic thromboembolic disease (CTEPH) and pulmonary hypertension secondary to heart failure. Additional exclusion criteria included the following: combination therapy, pregnancy, acute vasodilatory responses, peripheral vascular responses, parenchymal lung disease, patients refractory to first-line therapy, and management of pulmonary hypertension during or post-surgery.

### Statistical analysis

For each article that met the inclusion criteria, we calculated the placebo-corrected change from baseline for CI, mPAP and mRAP. If more than one trial was identified with the same therapeutic agent, the weighted, placebo-corrected change from baseline was calculated for that particular therapy. In order to determine an average baseline hemodynamic profile typical of patients enrolled in the RCTs, we calculated weighted means for CI, mPAP and mRAP for all patients randomized to placebo. Due to the variable nature of data reporting in the identified trials (e.g. mean ± SD, SEM, 95% confidence interval, range), weighted improvements are displayed as mean only.

To determine the estimated survival for placebo-treated patients, we substituted the baseline clinical profile from the identified trials into the equation developed by the National Institutes of Health (NIH) Registry [4]:

where H(t) = 0.88 - 0.14t + 0.01t^2 ^(t being the time in years), A(x, y, z) = EXP(0.007325x + 0.0526y - 0.3275z), x is mPAP, y is mRAP, and z is CI. The probabilities of survival at 1 year, 2 years, and 3 years are given by the following: P(1) = 0.75^A^; P(2) = 0.64^A^; and P(3) = 0.55^A^, respectively. The baseline clinical profile was then modified according to the benefit provided by each PAH-specific therapy and survival probabilities calculated at 1, 2 and 3 years.

## Results

The search yielded 54 potentially applicable references. After reviewing the title, abstract or text, 44 articles were excluded because of CTEPH [8,9], heart failure [10-14], combination therapy [15-18], acute vasodilatory responses [19,20], peripheral vascular responses [21,22], limited patient numbers [23], or the management of pulmonary hypertension during or post surgery [24-31]. Three articles were extensions or a substudy of a RCT [32-35]. Two articles were based on pharmacokinetic/dynamic differences [36,37], hemodynamic responses to altitude [38-40], and one article involved exercise training [41]. Eight studies were not placebo-controlled or did not provide sufficient hemodynamic data [42-49]. One was a dose ranging study [44], and one article returned by the search strategy was a review [50]. Finally, we reviewed 10 articles [2,51-59].

### Characteristics of studies

Additional file 1 presents an overview of the RCTs identified by the search. Suitable hemodynamic data were identified for bosentan, sitaxentan, sildenafil, epoprostenol, beraprost and treprostinil. Ambrisentan was not included due to the absence of suitable data. The mean trial duration was 18 ± 13 weeks (range: 12-52 weeks). The trials recruited 1,635 patients: 77.6% were female and the mean (SD) age of patients was 46.5 ± 4.9 years. A total of 908 patients (55.5%) had iPAH, 390 (23.9%) PAH related to CTD and 298 (18.2%) PAH related to CHD. PAH related to HIV and portal hypertension was present in 18 (1.1%) and 21 (1.3%) patients, respectively. The majority of patients included in the RCTs were in NYHA/WHO functional class III and the mean 6 MWD was 355.2 ± 56.3 m.

For the entire population, disease etiologies were generally similar between treatment groups. More patients with PAH related to CHD were included in the bosentan group (35%) compared to other treatments (6-29%). This was largely due to the inclusion of a RCT investigating bosentan exclusively in patients with Eisenmenger syndrome [55]. The baseline hemodynamics were relatively heterogeneous between groups, perhaps skewed slightly to patients with a more favourable hemodynamic profile in the bosentan group, and to more severely effected patients in the epoprostenol group. The characteristics of patients are listed in additional file 2.

### Effects of treatment of cardiopulmonary hemodynamics

According to the NIH Registry equation, the greatest influence upon survival is time itself, followed by changes in CI, mRAP and mPAP, respectively. Additional file 3 displays the weighted mean changes in right ventricular hemodynamics for each PAH-specific treatment and, based on these changes, the predicted hemodynamics post-therapy. Overall weighted improvements are small to moderate. Bosentan, epoprostenol and high-doses of sitaxentan and sildenafil provide the greatest improvements in CI: the variable with the greatest impact on survival. Intravenous epoprostenol provided the greatest reductions in mRAP and mPAP. Relative to other PAH-specific therapies, beraprost and low dose sildenafil delivered minimal improvements in hemodynamic parameters.

One-, 2- and 3-year survival estimates for each therapy are presented in table 1. The estimates are based on the NIH Registry equation and the predicted, post-therapy hemodynamics derived from the systematic review. Based on the effects observed in short-term trials and, relative to placebo, all therapies analysed appear to improve survival. In the first year, bosentan improves survival from 71.9% to 77.8%. Moreover, at year 3 the predicted survival without treatment would be 50.4%, while in the same patient treated with bosentan it would be 59.3%. Comparable survival trends were observed between epoprostenol and bosentan, and between sildenafil 20 mg and sitaxentan 100 mg.

**Table 1:** Predicted survival with PAH-specific therapy using weighted mean changes from baseline in RCTs.

	Predicted survival*		
Treatment	Year 1	Year 2	Year 3
**Placebo**	0.719	0.600	0.504
**Bosentan**	0.778	0.677	0.593
**Sitaxentan**			
**100 mg**	0.758	0.651	0.562
**300 mg**	0.757	0.649	0.560
**Sildenafil**			
**20 mg**	0.752	0.642	0.552
**40 mg**	0.756	0.648	0.559
**80 mg**	0.771	0.668	0.583
**Epoprostenol**	0.784	0.686	0.603
**Beraprost**	0.741	0.629	0.537
**Treprostinil**	0.761	0.655	0.567

*Based on the NIH Registry equation.

## Discussion

The treatment of PAH has realised dramatic advances over the past decade, and it is clear that the PAH-specific therapies can significantly alter the natural history of the disease. However, there remains a need to differentiate between the available therapies and a means to identify which treatment responses have the most prognostic value. Ideally, the best way to compare treatments is by head-to-head comparisons but, in the absence of such trials, the choice of treatment is currently dictated by a comparison of the available data and the logistics of drug availability. The results of our review show a consistent improvement in right ventricular hemodynamics with PAH-specific therapy, with small to moderate heterogeneity in achieving this effect. When applied to the NIH Registry equation, these improvements result in survival estimates superior to placebo-treated patients. However, using indirect comparisons with long-term follow-up studies, the accuracy with which the NIH Registry equation predicts survival for PAH therapies other than epoprostenol appears to be less than accurate.

In a broad cohort of PAH patients treated with bosentan, we calculated a predicted survival of 77.8%, 67.7% and 59.3% and 1-, 2- and 3-years, respectively. This estimate is considerably lower than that observed in several open-label and registry studies where 1-year observed survival with bosentan-treated patients ranges between 89.3% and 97% [60-62]. McLaughlin and Provencher both published survival data from open-labelled extension studies with patients involved in the pivotal bosentan clinical trials [60-62]. The mean follow-up period was greater than 2 years in both studies and 1, 2- and 3-year estimates of survival in the retrospective analysis by Provencher were 92%, 89% and 79%, respectively: considerably greater than the predicted survival rates observed in the present study. Similarly, our estimated survival with bosentan is well below that reported in a French National Registry published by Humbert et al. [63]. In that study, consisting of 674 adult patients, and in which epoprostenol or bosentan were available, the 1-year survival rate amongst patients with iPAH, familial or anorexigen-induced PAH was 89.3%.

Disagreement can also be found between the predicted survival with sitaxentan in our study, and that reported elsewhere in the literature. The Sitaxentan To Relieve Impaired Exercise (STRIDE)-2X open-label extension study recently reported 1-year observations of PAH patients treated with sitaxentan or bosentan [64]. Patients treated with sitaxentan had a 96% overall survival rate at 1-year compared to 88% in patients treated with bosentan. Both results are notably different to those estimated using the NIH Registry equation. It is important to recognize, however, that in making these indirect comparisons, the survival estimates associated with long-term, unblinded extension studies and patient registries are not necessarily attributable to the PAH-specific therapy alone. Increased surveillance and the addition of concomitant therapies may have contributed to the improved survival, compared with that estimated using the NIH equation. Comparisons between our data and long-term follow-up studies should therefore be treated with caution.

Our estimates of survival correspond more closely to those observed with epoprostenol. Sitbon et al. assessed long-term survival in 178 patients with iPAH between 1992 and 2001 [65]. Overall survival rates at 1-, 2- and 3-years were 85%, 70% and 63%, respectively. Similarly, a retrospective Scottish study conducted on patients between 1986 and 2001 estimated 1-year survival rates of 70-80%,[66] comparable to the results observed in our review (78.4%, 68.6% and 60.3%).

We noted in our study a range of survival estimates for the different PAH-specific therapies. The baseline difference in hemodynamics could by itself have influenced this outcome. However, we compared changes in all treatment groups against the same standardized clinical profile. Furthermore, it would be reasonable to expect that trials including patients with hemodynamic values closer to normal would display a smaller overall treatment effect, and conversely, trials including patients with the greatest degree of functional limitation to display the greatest overall gains. This was not the case. At baseline, the bosentan group displayed the best hemodynamic profile, yet still delivered one of the greatest improvements following therapy.

Although the methodology of the included RCTs was homogeneous, the patient populations were not. For example, one trial with bosentan focused solely on patients with Eisenmenger syndrome [55], and one trial exclusively assessed patients with functional class II disease [58]. It is known that the underlying diagnosis associated with PAH can influence outcomes [6]. However, we decided to include these articles because the registration trials for sildenafil[56] and sitaxentan[51] included patients with both CTD and CHD, and the majority of RCTs included patients with class II disease [51-53,56,57,59]. To exclude these trials would make comparisons between therapies difficult.

Our review of the data is consistent with the view that short-term changes in hemodynamics are a contributing factor, rather than the sole factor, in determining the long-term response to therapy. Indeed, the disagreement between predicted and real survival in patients with PAH, reflects the fact that other variables, not included in the NIH Registry equation, need to be identified. Further investigation of how PAH-specific therapies impact on right ventricle function itself may yield reward in understanding how the impact of therapy translates into a reduction of morbidity and mortality. No direct hemodynamic studies between selective and non-selective endothelin receptor antagonists exist in patients with PAH. However, an echocardiographic study by Galie et al., in 85 patients with PAH, demonstrated that bosentan therapy for 16 weeks improves right ventricular systolic function and early left ventricular diastolic filling leading to a decrease in right ventricular dilation and an increase in left ventricular size [67]. Together, these effects may explain in part, the improved survival observed in patients with PAH treated with bosentan.

There are several limitations of our analysis that warrant consideration. Firstly, the individual trials are too small to show statistical differences between the respective therapeutic groups. Secondly, we included RCTs performed over a 12 year period and over various time frames (12 to 52 weeks). We acknowledge that different treatment practices within this time may be a contributing factor on our results. Also, a publication bias favouring positive studies cannot be excluded. We included trials with compounds or doses of medications that were eventually not approved. However, we thought it necessary to present a complete overview of the literature on this topic. In addition, it should be noted that vasoreactivity testing was not consistently reported by the included trials. Finally, the results presented in table 1 are based on a comparison of outcomes derived from a theoretical equation developed by the NIH in patients with iPAH in the pre-epoprostenol era. The clinical studies discussed here contain mixed etiologies and it is unknown whether the NIH equation provides an adequate guide for patients with other forms of PAH.

## Conclusion

Our results show that both new and old treatments improve hemodynamic parameters and improve survival compared to placebo-treated patients. However, the clinical implications of these modifications are less evident. Short-term improvements in hemodynamics, when applied to the NIH Registry equation, appear to underestimate the survival benefits observed with long-term therapy. It is clear that the relationship between hemodynamic improvements and survival in patients with PAH requires further investigation in large scale clinical studies with adequate follow-up. Nonetheless, our report provides a novel description of the association between hemodynamic measurements and predicted survival in patients with PAH.

## Competing interests

PS and SS have received travel support from Actelion Pharmaceuticals Australia as well as honoraria for speaking and consulting engagements. GS has been an employee of Actelion Pharmaceuticals Australia. JW have received consulting fees from Actelion Pharmaceuticals Australia. BD has received consulting fees from Actelion Pharmaceuticals Australia and Gilead Sciences Pty Limited. EG has received research support from Actelion Pharmaceuticals Australia, CSL as well as honoraria for speaking and consulting engagements, and as a member of the Actelion, CSL and GSK Advisory Boards. The Heart and Lung Transplant Foundation of WA, of which EG is chair has received educational grants from Actelion and CSL. EG has also received travel support from Bayer-Schering the manufacturers of iloprost, Encysive Pharmaceuticals, the manufacturers of sitaxentan and GSK, the distributors of ambrisentan in Australia. AK has participated in clinical trials with Actelion Pharmaceuticals Australia, Myogen, Pfizer, Roche, Novartis, Wyeth, Ventracor, Scios, Heartware, GSK and Bayer. She has served on Advisory Boards for Actelion Pharmaceuticals Australia, Pfizer, CSL, Novartis, Roche, Ventracor, GSK and Bayer.

## Authors' contributions

BD wrote the first draft of the manuscript. All authors provided essential input into all revisions of the manuscript. All authors gave final approval of the version submitted for publication.

## Pre-publication history

The pre-publication history for this paper can be accessed here:

http://www.biomedcentral.com/1471-2261/10/9/prepub

## Supplementary Material

Additional file 1 Characteristics of RCTs comparing PAH treatments and placebo for changes in cardiopulmonary hemodynamics. Click here for file

Additional file 2 Characteristics of patients enrolled in RCTs comparing PAH treatments and placebo for changes in cardiopulmonary hemodynamics. Click here for file

Additional file 3 Weighted mean improvements in cardiopulmonary hemodynamics and predicted values post-therapy. Click here for file
