# Supplementary material for: Hemodynamics in pulmonary arterial hypertension (PAH): do they explain long-term clinical outcomes with PAH-specific therapy?
Source: BMC Cardiovasc Disord. 2010 Feb 22;10:9. doi: 10.1186/1471-2261-10-9 (PMC2841582; doi:10.1186/1471-2261-10-9)
Supplement: Additional file 1 — Characteristics of RCTs comparing PAH treatments and placebo for changes in cardiopulmonary hemodynamics. [file 1471-2261-10-9-S1.DOC]

**Additional file 1:** Characteristics of RCTs comparing PAH treatments and placebo for changes in cardiopulmonary hemodynamics.

| **Author (year)** | **n** | **Inclusion criteria** | **Intervention** | **Comparator** | **Study duration** |
| --- | --- | --- | --- | --- | --- |
| Channick et al. (2001)^54^ | 32 | iPAH and CTD  WHO III/IV  6MWT 150-500 m | Bosentan 62.5 mg bd for 4 wks, then 125 mg bd for 8 wks | Placebo | 12 weeks |
| Galie et al. (2006)^55^ | 54 | CHD  WHO III | Bosentan 62.5 mg bd for 4 wks, then 125 mg bd for 12 wks | Placebo | 16 weeks |
| Galie et al. (2008)^58^ | 185 | iPAH/CTD/CHD/HIV  WHO II | Bosentan 62.5 mg bd for 4 wks, then 125 mg bd for 22 wks | Placebo | 26 weeks |
| Galie et al. (2005)^56^ | 278 | iPAH and CTD  WHO II-IV  6MWT 100-450 m | Sildenafil  20 mg tid  40 mg tid  80 mg tid | Placebo | 12 weeks |
| Barst et al. (2004)^51^ | 178 | iPAH/CTD/CHD  WHO II-IV  No 6MWT limit | Sitaxentan  100 mg/day  300 mg/day | Placebo | 12 weeks |
| Galie et al. (2002)^57^ | 130 | iPAH/CTD/CHD/HIV  WHO II/III  6MWT 50-500 m | Beraprost  Median 80 µg 4x/day | Placebo | 12 weeks |
| Barst et al. (2003)^53^ | 116 | iPAH/CTD/CHD  WHO II/III  No 6MWT limit | Beraprost  Median 120 µg 4x/day | Placebo | 52 weeks |
| Barst et al. (1996)^2^ | 81 | iPAH  WHO III/IV  No 6MWT limit | Epoprostenol | Placebo | 12 weeks |
| Badesch et al. (2000)^52^ | 111 | CTD  WHO III/IV  6MWT >50 m | Epoprostenol | Placebo | 12 weeks |
| Simonneau et al. (2002)^59^ | 470 | iPAH/CTD/CHD  WHO II-IV  6MWT 50-450 m | Treprostinil | Placebo | 12 weeks |

6MWT, 6-minute walk test; CHD, congenital heart disease; CTD, connective tissue disease; HIV, Human Immunodeficiency Virus; iPAH, idiopathic pulmonary arterial hypertension; WHO, World Health Organisation.
