# Supplementary material for: Hemodynamics in pulmonary arterial hypertension (PAH): do they explain long-term clinical outcomes with PAH-specific therapy?
Source: BMC Cardiovasc Disord. 2010 Feb 22;10:9. doi: 10.1186/1471-2261-10-9 (PMC2841582; doi:10.1186/1471-2261-10-9)
Supplement: Additional file 2 — Characteristics of patients enrolled in RCTs comparing PAH treatments and placebo for changes in cardiopulmonary hemodynamics. [file 1471-2261-10-9-S2.DOC]

**Additional file 2:** Characteristics of patients enrolled in RCTs comparing PAH treatments and placebo for changes in cardiopulmonary hemodynamics.

|  | **Bosentan** | **Sitaxentan** | | **Sildenafil** | | | **Epoprostenol** | **Beraprost** | **Treprostinil** | **Placebo** |
| --- | --- | --- | --- | --- | --- | --- | --- | --- | --- | --- |
|  |  | **100 mg** | **300 mg** | **20 mg** | **40 mg** | **80 mg** |  |  |  |  |
| **No. of patients** | 151 | 85 | 75 | 71 | 70 | 79 | 97 | 125 | 233 | 720 |
| **Mean (SD) age, years** | 45 ± 8 | 45 ± 14 | 44 ± 12 | 47 ± 14 | 51 ± 15 | 48 ± 15 | 47 ± 9 | 44 ± 3 | 45 ± 10 | 46 ± 5 |
| **Female, %** | 74% | 85% | 75% | 71% | 70% | 79% | 85% | 75% | 85% | 74% |
| **No. (%) patients with:** | | | | | | | | | | |
| **iPAH** | 71 (47%) | 23 (42%) | 34 (54%) | 44 (64%) | 43 (64%) | 46 (65%) | 41 (42%) | 82 (66%) | 134 (58%) | 397 (55%) |
| **CTD-PAH** | 22 (15%) | 16 (29%) | 17 (27%) | 21 (30%) | 20 (30%) | 21 (30%) | 56 (58%) | 11 (9%) | 41 (17%) | 177 (25%) |
| **CHD-PAH** | 53 (35%) | 16 (29%) | 12 (29%) | 4 (6%) | 4 (6%) | 4 (6%) | 0 | 16 (13%) | 58 (25%) | 108 (15%) |
| **Baseline hemodynamics:** | | | | | | | | | | |
| **CI (L/min/m^2^)** | 2.6 ± 0.2 | 2.4 ± 0.8 | 2.3 ± 0.7 | 2.4 ± 0.7 | 2.3 ± 0.7 | 2.5 ± 0.8 | 2.0 ± 0.1 | 2.6 ± 0.2 | 2.4 ± 0.1 | 2.3 ± 0.2 |
| **mPAP (mmHg)** | 61 ± 14.2 | 54 ± 17 | 54 ± 14 | 54 ± 13 | 49 ± 13 | 52 ± 16 | 56 ± 7 | 57 ± 1 | 62 ± 1 | 56 ± 6 |
| **mRAP (mmHg)** | 7.6 ± 1.9 | 7.0 ± 5.0 | 9.0 ± 5.0 | 8.0 ± 5.0 | 9.0 ± 6.0 | 9.0 ± 5.0 | 13.1 ± 0.1 | 8.2 ± 0.2 | 10.0 ± 0.4 | 9.1 ± 2.0 |

CI, cardiac index; CHD, congenital heart disease; CTD, connective tissue disease; iPAH, idiopathic pulmonary arterial hypertension; mPAP, mean pulmonary artery pressure; mRAP, mean right atrial pressure; SD, standard deviation.
