# Supplementary material for: Hemodynamics in pulmonary arterial hypertension (PAH): do they explain long-term clinical outcomes with PAH-specific therapy?
Source: BMC Cardiovasc Disord. 2010 Feb 22;10:9. doi: 10.1186/1471-2261-10-9 (PMC2841582; doi:10.1186/1471-2261-10-9)
Supplement: Additional file 3 — Weighted mean improvements in cardiopulmonary hemodynamics and predicted values post-therapy. [file 1471-2261-10-9-S3.DOC]

**Additional file 3:** Weighted mean improvements in cardiopulmonary hemodynamics and predicted values post-therapy.

|  | **Treatment effect** | | | | **Predicted hemodynamics values post-therapy** | | |
| --- | --- | --- | --- | --- | --- | --- | --- |
| **Therapy** | **No. studies (patients)** | **mPAP**  **(mmHg)** | **CI**  **(L/min/m^2^)** | **mRAP**  **(mmHg)** | **mPAP**  **(mmHg)** | **CI**  **(L/min/m^2^)** | **mRAP**  **(mmHg)** |
| **Bosentan** | 3 (254) | - 5.8 | +0.5 | -1.2 | 50.2 | 2.8 | 7.9 |
| **Sitaxentan**  **100 mg**  **300 mg** | 1 (178) | - 3.0  - 5.0 | +0.3  +0.4 | -1.0  0 | 53.0  51.0 | 2.6  2.7 | 8.1  9.1 |
| **Sildenafil**  **20 mg**  **40 mg**  **80 mg** | 1 (278) | - 2.7  - 3.2  - 5.3 | +0.2  +0.2  +0.4 | -1.1  -1.4  -1.3 | 53.3  52.8  50.7 | 2.5  2.5  2.7 | 8.0  7.7  7.8 |
| **Epoprostenol** | 2 (192) | - 6.3 | +0.4 | -2.4 | 49.7 | 2.7 | 6.7 |
| **Beraprost** | 2 (246) | - 1.5 | +0.1 | -1.0 | 54.5 | 2.4 | 8.1 |
| **Treprostinil** | 1 (470) | - 3.0 | +0.2 | -1.9 | 53.0 | 2.5 | 7.2 |

CI, cardiac index; mPAP, mean pulmonary artery pressure; mRAP, mean right atrial pressure. Data are means only.
